# The complete mitochondrial genome of a marine gastropod: *Umbonium thomasi* (Trochida, Trochidae)

**DOI:** 10.1080/23802359.2018.1535864

**Published:** 2018-11-25

**Authors:** Hana Kim, Cheol Yu, Hyung June Kim, Yun-Hwan Jung

**Affiliations:** a Department of Taxonomy and Systematics, National Marine Biodiversity Institute of Korea, Seocheon-gun, Chungcheongnam-do, Republic of Korea;; b Department of Biological Sciences, Inha University, Incheon, Republic of Korea;; c Department of Oceanography and Ocean Environmental Sciences, Chungnam National University, Daejeon, Republic of Korea

**Keywords:** Mitochondrial genome, *Umbonium thomasi*, Trochidae, marine gastropod

## Abstract

The mitogenome sequence of a marine gastropod, *Umbonium thomasi* (Crosse, 1863) (Trochida, Trochidae), was determined first in the genus *Umbonium*. The assembled mitogenome, consisting of 15,998 bp in length, has 13 protein-coding genes, 22 transfer RNA genes, and two ribosomal RNA genes. The order and structure of the genes are similar to those of other Trochid species. The overall base composition of mitogenome is 34.8% A, 22.1% C, 14.0% G, and 29.1% T with an A + T bias of 64%. The complete mitogenome of *U. thomasi* provides essential and important DNA molecular data for further phylogenetic and evolutionary analysis of marine gastropods.

*Umbonium thomasi* (Crosse, 1863) (Gastropoda, Trochida, Trochidae) are comparatively easy discovery on the coast of Korea, China, and Japan (Park et al. 1998; Lee 2016). Genus *Umbonium* plays an important role in the marine ecosystem. Genus *Umboinum* includes *U. thomasi*, which is the widely used food source for fish and shell borer gastropod, and are considered to affect the distribution of near organism in the marine ecosystem (Berry 1982; 1984; Park et al. 1998). Despite its importance, a study on genus *Umbonium* was only population dynamics based, yet our understanding of their genetics is limited. In this study, we have determined and described the complete mitochondrial genome (mitogenome) of *U. thomasi* for the first time.

The specimen was collected from sandy shore adjacent to the Yubu-do island in the Yellow Sea of Korea. The voucher specimen was deposited in the National Marine Biodiversity Institute of Korea (MABIK MO00171429). The genomic DNA was extracted from the muscle tissue by DNeasy Blood & Tissue Kit (Qiagen, Hilden, Germany) according to the manufacturer’s protocol. The mitogenome sequences were analyzed by next-generation sequencing in the Illumina Hiseq2000 sequencing platform (Macrogen, Seoul, Korea). These sequences were annotated in comparison with previously reported mitogenome sequences of Trochid species (Lee et al. 2016; Wort et al. 2017) using Geneious 9.1.8 (Biomatters Ltd, Auckland, New Zealand) (Kearse et al. 2012). Additionally, we used the mitochondrial genome annotation (MITOS) server (Bernt et al. 2013) and tRNAscan-SE server (Lowe and Chan 2016) for annotation. Maximum-likelihood (ML) tree was constructed to investigate the molecular taxonomic position of these species using GTR + G model in MEGA6 (Tamura et al. 2013) and dataset used were nucleotide sequences of 13 protein-coding genes (PCGs) from the mitogenomes of the other 12 species in the order Trochida.

The circular mitogenome of *U. thomasi* (GenBank accession number MH729882) is 15,998 bp in length and has 13 PCGs, two ribosomal RNA genes (rRNAs), and 22 transfer RNA genes (tRNAs). The gene arrangement and structure of the genes are similar to those of other Trochid species. The overall base composition of the genome is 34.8% A, 22.1% C, 14.0% G and 29.1% T, exhibiting an A + T bias (64%). The 12 PCGs (*cox3*, *nad3*, *nad2*, *cox1*, *cox2*, *atp8*, *atp6*, *nad4*, *nad4l*, *cytb*, *nad6*, and *nad1*) get off by the typical ATG start codon, but one (*nad5*) use ATT as the initiation codon. The nine PCGs (*nad3*, *nad2*, *cox1*, *cox2*, *atp8*, *atp6*, *nad4*, *cytb*, and *nad1*) use TAA as the stop codon and four PCGs (*cox3*, *nad5*, *nad4l*, and *nad6*) ends with TAG. The lengths of tRNAs range from 64 to 70 bp and all tRNAs have the typical clover leaf structure.

In the ML tree of concatenated nucleotide sequences from 13 PCGs, *U. thomasi* was clustered together with other species within Trochidae with a high bootstrap value of 100% and is clearly separated from each genus (Figure 1). The mitogenome of *U. thomasi* will be useful for inferring the phylogenetic relationships among the members of Trochidae within the Trochida.

**Figure 1.**
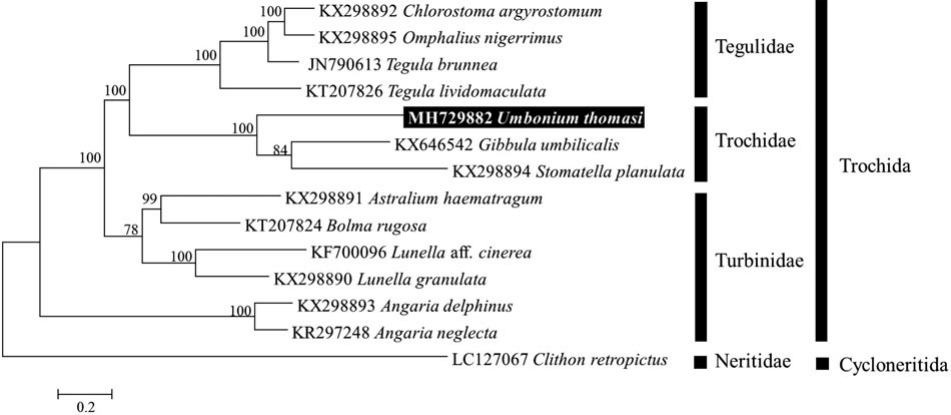
Maximum-likelihood (ML) tree based on the protein-coding genes (PCGs) of *Umbonium thomasi* with family Trochidae and other gastropod under order Trochida. *Clithon retropictus* derived from Cycloneritida was used as outgroup for tree rooting. Numbers above the branches indicate ML bootstrap values from 1000 replications.
